# Clinical Service Incorporating Mobile Technology on Weight Loss in Patients With Metabolic Dysfunction–Associated Steatotic Liver Disease: A Translation From Research Trial

**DOI:** 10.1002/edm2.485

**Published:** 2024-04-29

**Authors:** Siew Min Ang, Su Lin Lim, Yock Young Dan, Yiong Huak Chan, Qai Ven Yap, Juliana Chen

**Affiliations:** ^1^ Department of Dietetics National University Hospital Singapore Singapore; ^2^ Department of Medicine National University Hospital Singapore Singapore; ^3^ Biostatistics Unit, Yong Loo Lin School of Medicine National University Singapore Singapore Singapore; ^4^ Susan Wakil School of Nursing and Midwifery, Faculty of Medicine and Health, Charles Perkins Centre The University of Sydney Sydney New South Wales Australia

**Keywords:** nonalcoholic fatty liver disease, telemedicine, translational research

## Abstract

**Background:**

The prevalence and healthcare cost of metabolic dysfunction–associated steatotic liver disease (MASLD) has increased alongside the epidemic surge in obesity and Type 2 diabetes. Weight loss through lifestyle modification remains the primary effective therapy for MASLD. Incorporation of mobile technology in lifestyle interventions has been previously found to be efficacious and cost‐effective in facilitating weight loss. However, there is a paucity of studies that have successfully translated lifestyle research into clinical service for weight loss to alleviate disease burden. Our study aimed to describe the process of translating a mobile technology–enabled trial into a tertiary hospital outpatient dietetics service for patients with MASLD.

**Methods:**

The Iowa Model of Evidence‐Based Practice to Improve Quality Care was used as a framework for this paper to guide implementation at the organizational level.

**Results:**

Regular engagement of key operational staff and the hospital management team facilitated open discussions of the challenges faced and enabled rapid implementation of strategies that contributed to the smooth piloting of the service. A service adoption rate of 81% was achieved. Preliminary outcome evaluation found that the percentage of patients achieving ≥ 5% weight loss from baseline at 6 months was comparable at 54% and 52% for the service and trial groups, respectively.

**Conclusions:**

Evaluation of the implementation process found that a hybrid model of care (in‐person consultation supplemented with app coaching) preserved interpersonal connections while maximizing the convenience and scalability of mobile app–enabled service. Although high digital acceptance and adoption rates propelled by COVID‐19‐supported telehealth, it is prudent to assess patient's access to technology and digital literacy and offer resources to help them benefit from telehealth services.

## Graphical abstract

Adapted version of the Iowa Model of Evidence‐Based Practice to Improve Quality Care framework, illustrating the process of successfully translating a mobile technology–enabled research trial into a clinical dietetic service for patients with metabolic dysfunction–associated steatotic liver disease.

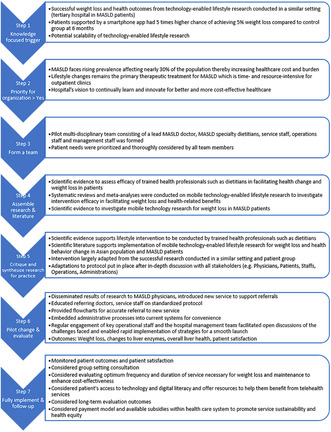

Metabolic dysfunction–associated steatotic liver disease (MASLD) is recognized as the leading cause of chronic liver disease and its complications, with prevalence affecting 30% of the global population [1, 2]. The economic burden of MASLD is significant, with an annual direct medical cost estimated to be USD103 billion and €35 billion in the United States and Europe‐4 countries, respectively, in 2016 [3]. Prevalence of the disease is rapidly increasing alongside metabolic syndrome, Type 2 diabetes and obesity [2]. It has been reported that 60%–80% of individuals who have diabetes or obesity are diagnosed with MASLD [2]. The strong link between MASLD and metabolic syndrome, characterized by insulin resistance, makes it crucial to increase disease awareness, address cardiometabolic risk factors and offer treatment for MASLD with an all‐rounded approach [4, 5]. Although a growing number of pharmacotherapy options are available for managing obesity and comorbidities, which could potentially improve MASLD, there are currently no approved medications specifically for MASLD. Bariatric surgery may be considered as a treatment option; however, only patients who meet the criteria for metabolic surgery and can afford the cost would potentially benefit [5, 6]. For most patients with MASLD, aiming for a 7%–10% reduction in body weight can effectively reduce cardiometabolic risk factors, hepatic steatosis and improve liver health [4, 5, 7]. Weight loss through intensive lifestyle modifications remains the primary effective therapy [2, 5]. In more recent years, the utilization of mobile technology as part of lifestyle intervention has gained traction because of the convenience, ubiquity and scalability of smartphone applications (apps) for researchers to support patients remotely. With greater opportunities for communication, patient and healthcare professional relationship is enhanced, leading to better health outcomes [8, 9]. Overall, systematic reviews have concurred that incorporating mobile apps in lifestyle interventions for weight loss is efficacious in the short to medium term and are cost‐effective [8, 9, 10].

With positive results from mobile technology–enabled lifestyle interventions, translating it from research to the real‐world setting to alleviate disease burden appears promising [7]. However, there is a paucity of studies that have successfully translated mobile technology–enabled lifestyle research into clinical service for weight loss. Research on lifestyle interventions is typically conducted in highly controlled settings with motivated participants, adequate funding and well‐trained professionals to deliver the programme [11, 12]. These optimal conditions for intervention delivery are vastly different from those observed in clinical settings in which patients are highly varied, time is limited, and resources are constrained [12].

## 1 Aim

Therefore, the aim of this paper was to describe the process of translating a mobile technology–enabled trial into a tertiary hospital dietetics clinical service for patients with MASLD. We have used the Iowa Model of Evidence‐Based Practice to Improve Quality Care as a framework for this paper [13, 14], allowing us to demonstrate implementation of best research evidence into clinical practice at the National University Hospital (NUH) Singapore. The Iowa Model is a seven‐step process: (1) Knowledge‐focused trigger, (2) Priority for organization, (3) Form a team, (4) Assemble literature for practice, (5) Critique and synthesize literature for practice, (6) Pilot change and evaluate and (7) Fully implement and follow‐up.

### 1.1 Step 1—Knowledge‐Focused Trigger

Lim et al. [15] conducted a mobile technology–enabled MASLD‐specific lifestyle randomized controlled trial in 2017. The trial was efficacious in facilitating weight loss and clinical improvements in a tertiary hospital setting. Patients who were supported by dietitians via a smartphone app had five times greater chance of achieving clinically significant 5% weight loss than control group patients at 6 months. Furthermore, this mobile technology–enabled trial was reported to be potentially scalable [15]. This knowledge‐focused trigger if translated into a clinical setting could have the potential to increase provider to patient ratio with standardized training, protocol and dissemination of resources and information [7, 15]. In addition, the use of a smartphone app could allow patients to receive nutrition and lifestyle intervention, monitoring and evaluation from their healthcare providers in a timely and remote fashion that would improve their weight and health outcomes [16].

### 1.2 Step 2—Priority for Hospital

As MASLD prevalence increases, the demand for MASLD‐specific outpatient services have grown, leading to greater strain on existing resources [17]. With a vision to continually learn and innovate for better and more cost‐effective health care, NUH recognized the potential and priority of translating research into clinical services in a value‐based manner. As mobile health intervention has been found to be cost‐effective [10], the hospital supported the launch of the mobile technology–enabled dietetics clinical service.

### 1.3 Step 3—Form a Team

The National Institutes of Health (NIH) Roadmap for Research Teams of the Future recommends that a collaborative approach that engages all stakeholders can increase the success of translating research to service [11]. Consequently, a pilot multidisciplinary team was formed, comprising of MASLD doctors, dietitians, service staff, operations staff and management staff, to facilitate the launch. Although there was no patient representative on the team, patient needs were prioritized and thoroughly considered by all team members. Throughout the planning phase of the service, stakeholder needs were discussed extensively by the team to adapt the original research trial into an acceptable and realistic outpatient service. The regular team meetings facilitated discussion of challenges faced and enabled the rapid implementation of strategies for a smooth launch.

### 1.4 Steps 4 and 5—Assemble, Critique and Synthesize Research and Literature for Practice

Traditionally, patients with MASLD in NUH received outpatient care consisting of general lifestyle advice offered by a trained nurse. However, examination of the scientific literature found that trained health professionals such as dietitians are effective in facilitating health changes and achieving weight loss [15, 18]. Besides being clinically effective, a review by Howatson, Wall, and Turner‐Benny [19] also reported dietetic interventions being cost‐effective compared with physician‐only care. It has been found that dietitians are utilizing mobile apps as an information resource with patients and for patient self‐monitoring, but apps are less commonly used for behaviour change [20]. Our previous systematic review and meta‐analysis also showed that in the Asian population, weight loss is enhanced when routine care is supplemented with mobile app [9]. Therefore, the literature supports implementing mobile technology in dietetic counselling to facilitate weight loss in patients with MASLD.

Translation of Lim et al.'s MASLD‐specific trial to clinical service is outlined in Table 1. This service largely adopted the research intervention protocol but included modifications necessary to create a sustainable service delivery model, including a fee‐paying clinical service structure. The clinical service retained the hybrid model of an in‐person initial consultation supported by interactive remote coaching on a smartphone for a month. Patients were then provided the option to pay and extend the app‐coaching service after the first month, rather than just receiving it as part of the intervention for 6 months as per the original trial. The first visit offered healthy lifestyle education, individualized recommendations, goal setting, app set‐up and an app familiarization tutorial in accordance with trial protocol. The smartphone app, Nutritionist Buddy (nBuddy), utilized in the trial was retained for the clinical service as it was multi‐functional and culturally adapted to the local population (see Figure S1) [21, 22, 23]. Key app features include weight, diet and physical activity tracking. Automated feedback on excess oral intake and education on healthier alternatives were available to help patients adhere to recommendations. Personalized feedback and encouragement from dietitians were offered via the interactive chat channel in the app daily for the first week to engage patients. An average of 10 min would be spent coaching each patient. Coaching was tapered to around two to three times a week in the following weeks. All questions on the interactive chat were answered within one working day to maintain engagement.

**TABLE 1:** Translation of the research trial into a hospital outpatient clinical service.

	Research trial^a^ (January–June 2018)	Dietetics clinical service incorporating mobile technology (June 2018–June 2021)
Patient demographic	Overweight or obese MASLD, aged 21–70 years Controlled for various parameters, stratified by gender, age and BMI	All who were referred from gastroenterology doctors Patients were aged 21–80 years
	Screening done by research team according to inclusion criteria: Adults aged ≥ 21 years, BMI ≥ 23 kg/m^2^, proficient in English language and owns a smartphone	Brief screening done by gastroenterology doctors—inclusion criteria: proficient in English language, owns a smartphone and willing to use app for health coaching
	Patients were also stratified according to BMI range and gender	There were no restrictions on BMI or gender to the clinic
	Exclusion: Exceeding the recommended alcohol intake of 1.5 times, hepatitis, cirrhosis, poorly controlled diabetes, recent cardiovascular event, Stage 4 kidney disease, depression and pregnant women	Minimal exclusion criteria
Intervention	Duration: 6 months app coaching	Duration: 1 month app coaching with an option to extend via chargeable monthly app coaching
	First visit: Provided dietary assessment Educated on dietary and physical activity modifications Set individualized weight and lifestyle goals Downloaded and taught how to use nBuddy mobile app Weight, waist circumference, blood pressure and blood tests (LFT, FBG and HbA1c) taken Offered weighing scale Two optional workshops over course of 6 months First diet counselling provided by research dietitians Subsequent coaching via the app provided by the research dietitians	First visit: Provided dietary assessment Educated on dietary and physical activity modifications Set individualized weight and lifestyle goals Downloaded and taught how to use nBuddy mobile app Weight, blood pressure and blood tests (LFT, FBG and HbA1c) taken as part of outpatient visit (blood test subjected to doctor orders)
		First diet counselling provided by clinical dietitians Subsequent coaching via the app provided by the clinical dietitians
	Consultations and coaching via the app provided by research dietitians	Consultations and coaching via the app provided by clinical dietitians
Reviews/Follow‐up	Standardized at 3‐month, 6‐month	Review at 3–4 months in clinic
	Bookings of appointment handled by research team	Bookings of appointment handled by patient service staff (ideally fixed on the same day as doctor follow‐up visit for patient's convenience)
	Review visits: Weight, waist circumference, blood pressure taken Blood tests (LFT, FBG and HbA1c) taken Progress review and tailored lifestyle recommendations offered	Review visits: Weight and blood pressure taken Blood tests (LFT, FBG and HbA1c) taken as part of outpatient visit subjected to doctors' orders Progress review and tailored lifestyle recommendations offered
Fees and charges	Nil charges, fully covered by research funds	Full consultation fees charged to patients. Optional monthly extension of dietitian app coaching payable by patients. Fees for the first visit ranged from SGD40 to 150 whereas app‐coaching extensions were charged between SGD20 to 75 per month. The exact amount that patients were required to pay was determined by the level of subsidy that they receive from the Singapore government for public health care
	Administrative work handled by research assistant	Administrative work handled by patient service staff

Abbreviations: BMI, body mass index; FBG, fasting blood glucose; HbA1c, glycated haemoglobin; LFT, liver function tests; MASLD, metabolic dysfunction‐associated steatotic liver disease.

^a^ Lim et al. [15].

### 1.5 Step 6.1—Pilot the Change

**FIGURE 1:**
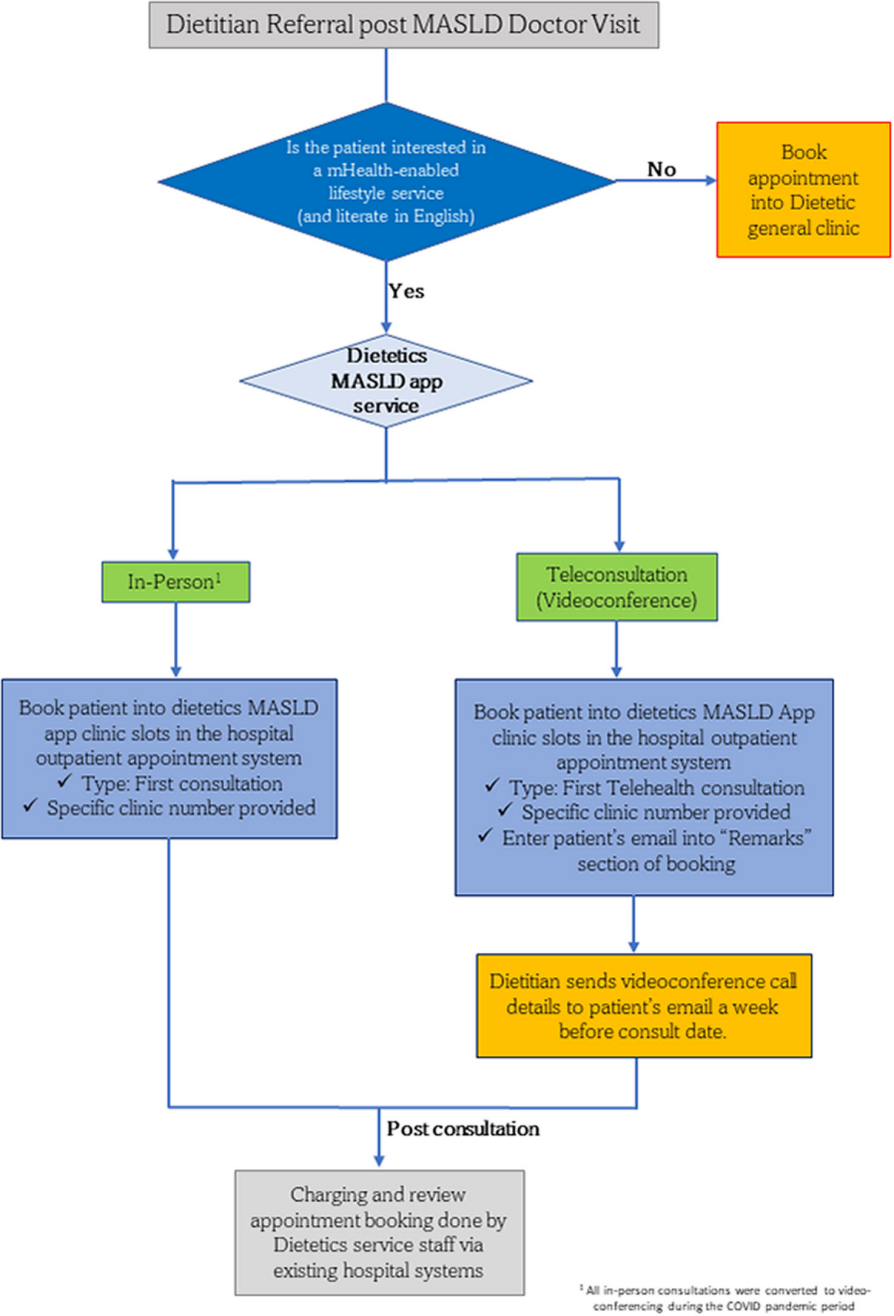
Flow chart illustrating the referral and appointment booking process of patients with MASLD into the dietetics MASLD app service clinic.

Doctors were briefed that a new evidence‐based clinical service for patients with MASLD had been piloted which included a dietitian consultation supplemented with interactive remote support via a smartphone app. They were encouraged to share and refer patients to the service. A clinical workflow (Figure 1) was developed and circulated to assist service staffs from the MASLD clinic in booking patients into the appropriate dietetics' specialty clinic. The initial dietetics consultation for the new service was scheduled to run on the same day as the MASLD clinic to promote service uptake. At the start of the dietetics consultation, all patients were explained the benefits and costs of the mobile app–coaching service by their dietitians and offered a choice to uptake the app‐coaching service. Additional resources and step‐by‐step guide for the app familiarization tutorial were developed specifically for the session to reduce barriers to adoption. After attending the service, patients would be prompted to book a review session and make payment in the same way as a regular outpatient appointment. The fees for the first consultation ranged from SGD40 to 150. Patients who did not uptake the app‐coaching service paid between SGD30 to 115 for their consultation. The exact amount payable was determined by the level of subsidy that patients receive from the Singapore government for public health care [24]. Enhancing health equity among MASLD patients with lower socioeconomic status could potentially increase their health literacy and encourage healthier lifestyle choices, thereby offering them a better chance to improve their liver and metabolic health [6].

During the COVID‐19 pandemic, the in‐person consultation was replaced by videoconferencing. Phone calls prior to the consultation were made by dietitians to assess whether patients were able to set up and join the videoconferencing session. Review appointment booking and billing were done virtually in accordance with hospital practice.

### 1.6 Step 6.2.1—Evaluate the Change

A retrospective chart audit was conducted on a cohort that accessed the mobile technology–enabled dietetics service between June 2018 and 2021 at NUH. This study was approved by the Domain Specific Review Board of the National Healthcare Group (NHG DSRB Ref.: 2021/00663). Patients were referred by gastroenterologists at the NUH outpatient MASLD clinic. All patients who were able to communicate in English and owned a smartphone were eligible for service.

Anthropometric measurements and biochemical tests were taken by trained hospital staff as part of routine monitoring at the MASLD clinic. Self‐reported body weight on the app was used when a weight was unavailable in the medical record system. Patients' baseline demographics such as gender, age, ethnicity, liver elastography score, alcohol consumption and existing medical comorbidities were also collected. A patient satisfaction survey was also disseminated to obtain feedback on the mobile technology–enabled dietetics service provided. Patient data were de‐identified by the Research Office Central Trusted Thirty Party before use.

Data analyzes were performed using the software package IBM SPSS for Windows version 29. Multiple imputations method was used to derive missing data points, with five imputations performed for each missing value using the Markov chain Monte‐Carlo method. The results from five imputed data sets were combined. Descriptive statistics were summarized as mean and standard deviation for normally distributed variables otherwise median and interquartile range were reported. Categorical data were presented as number and percentage. Logistic regression was performed on percentage weight loss at end of app‐coaching period, adjusting for age, gender, ethnicity, liver elastography score and alcohol consumption. Statistical significance was set at two‐sided *p* < 0.05.

### 1.7 Step 6.2.2—Preliminary Outcome Evaluation

A total of 109 patients attended the mobile technology–enabled lifestyle service and completed the 1‐month app‐coaching support. All patients referred for the service owned a smartphone. The adoption rate for the service was 81%; those who did not take up the additional app‐coaching service were mostly non‐English speakers. The mean age was 48.6 years, and 49% were female. Regular alcohol consumption was observed in 5.5% of the patients. The sample included a multiethnic population of 81% Chinese, 13% Malay and 3% Indian, which is representative of the ethnic distribution of the country [25]. See Table 2 for baseline patient demographics.

**TABLE 2:** Baseline demographics of study patients.

Variables	
Gender	
Female, *n* (%)	53 (48.6)
Male, *n* (%)	56 (51.4)
Ethnicity	
Chinese, *n* (%)	89 (81.7)
Malay, *n* (%)	14 (12.8)
Indian, *n* (%)	3 (2.8)
Others, *n* (%)	3 (2.8)
Alcohol consumption	
Yes, *n* (%)	6 (5.5)
No, *n* (%)	68 (62.4)
Social, *n* (%)	35 (32.1)
Age (years), mean (SD)	48.6 (12.0)
Fibrosis E score (kPa), median (IQR)	8.4 (6.6–10.8)
Comorbidities	
Diabetes, *n* (%)	35 (32.1)
Prediabetes, *n* (%)	24 (22.0)
Hyperlidaemia, *n* (%)	83 (76.1)
Hypertension, *n* (%)	54 (49.5)
Weight (kg), mean (SD)	79.5 (69.1–97.0)
BMI (kg/m^2^), mean (SD)	29.6 (26.9–33.0)
AST (IU/L), mean (SD)	40.0 (32.0–58.0)
ALT (IU/L), mean (SD)	59.6 (39.7–93.1)

Abbreviations: ALT, alanine aminotransferase; AST, aspartate aminotransferase; BMI, body mass index.

All patients underwent a standard of 1‐month app coaching as part of the initial dietetics consultation while 24/109 (22%) paid for app‐coaching extensions. Of the patients who received the standard 1‐month app coaching, 16.5% of them achieved ≥ 5% weight loss at 6 months. When app coaching was extended to 6 months, 52% achieved the target, showing that weight loss increases with app‐coaching duration (Table S1). In fact, we found patients who received ≥ 6 months of coaching were 11 times (adjusted odds ratio: 11.0, 95% CI: 2.5–48.3, *p* value = 0.001) more likely to achieve ≥ 5% weight loss compared to patients with less than 6 months of coaching (see Table S2). Both aspartate aminotransferase (AST) and alanine aminotransferase (ALT) levels showed improvement from baseline (see Table S1), but no clear trend was observed with a longer app‐coaching period.

All patients indicated satisfaction with the dietitian consultation (score range = 8–10 [most satisfied]), and 90% were satisfied with app coaching (score range = 7–9). Some responders complimented the personalized coaching helped them stay accountable. Patients mostly reported taking up the service to receive knowledge, support and accountability for weight loss and a healthy lifestyle to improve their liver condition. Feedback on service improvement were mainly technical, such as making meal logging easier, increasing the food database and offering more fitness‐related components.

### 1.8 Step 7—Using Pilot Evaluation to Inform Full Implement and Follow‐Up

This study is the first to illustrate a case study on a successful translation of a lifestyle research incorporating mobile technology for weight loss into a fee‐paying clinical service in patients with MASLD. The translation model presented in this study may guide healthcare providers, hospitals and other stakeholders with the framework for implementing research into other metabolic‐related services, including diabetes and weight management.

There is comparability in the outcomes patients achieve from the service and previous trial, with 54% and 52% of patients achieving ≥ 5% weight loss from baseline at 6 months, respectively. Another systematic review found 15%–44% of patients achieved 5% weight loss after receiving mobile app support for a mean duration of 18 weeks [9]. The levels of AST and ALT showed improvements with the reduction doubling when app coaching lasted at least 6 months. Patients who had lost ≥ 5% weight loss at 6 months were also more likely to show a reduction in their AST and ALT levels, indicating possible improvement in MASLD severity. The finding that patients have a higher odds of achieving ≥ 5% weight loss when engaging in at least 6 months of app coaching is consistent with another weight loss clinical service evaluation in which there was a 78% higher probability of patients achieving ≥ 5% weight loss with every additional 6 months of support [26]. Overall, results of the service and previous trial appeared comparable, which is a possible sign of a well‐translated research trial to clinical service.

This clinical service implemented a hybrid model of care, with in‐person consultation supplemented by remote monitoring and support for patients on a localized and multifunctional smartphone app. This model‐of‐care leveraged technology to relieve patients of increased costs and time burden associated with repeat consultations required for intensive lifestyle programmes, consistent with recommendations in the literature [8, 27, 28]. Migrating the physical sessions online allowed patients to access information at their own pace, self‐monitor lifestyle behaviours conveniently and contact their healthcare professional easily on the app. Meanwhile, the retention of the in‐person initial visit was crucial for setting goals and expectations, providing individualized care, building rapport and enhancing digital literacy. The human interaction is seamlessly transitioned onto the app by having the same dietitians in the clinic offer the app coaching to patients ensuring continuity of care. The inclusion of a qualified in‐app health professional may have contributed to a greater degree of self‐monitoring and adherence to lifestyle advice, thus facilitating weight loss in patients. Similarly in several reviews, it was found that lifestyle coaching by credible health professional increases engagement and adherence to recommendations which in turn, promotes weight loss [9, 29, 30]. For patients who have low app use, motivating and re‐engaging them on the app or scheduling in‐person reviews may be necessary. Overall, satisfactory experience and feedback reported by patients affirmed the quality of service rendered and fulfilment of health outcomes.

From a provider standpoint, the hybrid model research was realistically translated into a dietetics clinical service by embedding it into the existing hospital outpatient setting. By weaving key operational requirements from the research intervention into the existing hospital systems, medical documentation was easily shared and appointment booking and billing processes were seamlessly implemented. Streamlining in‐person consultations while providing lifestyle coaching digitally suits the time‐ and resource‐constrained outpatient setting. The acceptability of smartphone‐enabled interventions is likely to increase as global smartphone adoption grows to 73% by 2030 [31]. Therefore, mobile technology–enabled clinical services are a promising avenue to reduce the healthcare burden of MASLD efficiently and effectively.

It is noteworthy that the app‐coaching period was shortened from 6 months to 1 month with the option to extend to increase service adoption rates. A minimum period of 1 month was selected as app engagement typically drops after 2–3 months of use [7, 32]. This was decided by the team to reduce cost and commitment barriers for patients while providing flexibility for the app‐coaching duration. Although patients had to pay for the app‐coaching extensions, this allowed them to decide on the duration and may encourage autonomy over their health. This was supported by our finding that patients who extended the app‐coaching service for at least 6 months increased their likelihood of achieving the clinically significant 5% weight loss by 11‐fold. Attrition rates are common in clinical setting because of issues such as patient access, expectations and financial concerns [16]. Therefore, it is advisable to qualitatively evaluate the reasons for attrition and address them accordingly to retain patients. Future studies may also consider analyzing the cost‐effectiveness of translated services from research to provide insights to value‐based health care. For example, a study assessing the patterns of self‐monitoring on mobile app reported that long‐term intermittent monitoring may facilitate greater weight loss [33]. Providers could achieve greater cost‐effectiveness of the service by investigating and defining the optimum frequency and duration of app coaching needed for weight loss and maintenance. Furthermore, exploring the specific factors (e.g., app usage, engagement and lifestyle behaviour change) contributing to weight loss in multicomponent services may also help clinicians optimize outcomes while streamlining processes.

With the accelerated digitalization of multiple essential services during the COVID‐19 pandemic, patients have become more open to telemedical consultations and lifestyle coaching which worked in favour of our service. On the organizational level, a well‐supported digital infrastructure and a widely adopted telehealth communication software can facilitate the uptake of a health service [34]; ensuring that patients who are at increased risk of morbidity and mortality from COVID‐19 because of cardiometabolic diseases such as MASLD and diabetes can continue to receive treatment. Besides infrastructural support, providers were also geared towards telehealth through standardized training and telehealth guidelines. The standard of care provided was hence maintained and kept aligned with safety measures and data protection policy. The observation from our clinical service aligns with evidence that indicates that telehealth‐delivered dietetic services are comparable to traditional face‐to‐face care in the outcomes delivered and quality of service provided [35].

This study is not without limitations. Patients were recruited into the study on the basis of their autonomous adoption of the mobile app–coaching service and the higher initial cost associated may have potentially introduced a selection bias into the study. However, the tiered‐subsidy scheme introduced by the Singapore government would have provided greater cost reduction for patients requiring more financial support and enabled them to uptake healthcare services more readily. Although some non‐English speakers may have hesitated to adopt the English‐language mobile app service, most patients referred demonstrated a willingness and ability to use the app, as evidenced by the high adoption rate.

Key Lessons Learnt for Full Implementation

- Real‐life clinical services are associated with variable follow‐up periods, and it is recommended to schedule regular follow‐ups for monitoring.
- Greater cost‐effectiveness of the service could be achieved by investigating and defining the optimum frequency and duration of app coaching, as well as the specific factors (e.g., app usage, engagement and lifestyle behaviour change) necessary for successful weight loss and maintenance.
- Providers should assess for factors such as the patient's access to technology and digital literacy before pushing for telehealth in patients. Additional resources such as digital guidance and technical support should be supplemented to help patients benefit from technology–enabled health services.
- Despite the service being piloted in a MASLD‐specific patient group in a tertiary hospital outpatient setting, the outcomes and lifestyle intervention are highly applicable and could be translated across all other obesity and cardiometabolic disease management areas and healthcare settings.
- When translating the process to other countries, stakeholders should consider the payment model and available subsidies within the healthcare system. This adaptation is crucial for ensuring the service's sustainability and promoting health equity.
- Services with interventions lasting longer than a year will also be valuable to determine the effectiveness of mobile technology–enabled interventions in facilitating weight loss in the longer term.

This case study of a successful translation of mobile technology–enabled research into clinical service serves as a model for other researchers and healthcare services to adopt. The successes of the translated service relied on the hybrid model of care in which interpersonal connections were preserved while maximizing the convenience and scalability of mobile app–enabled services. Regular engagement with key operational staff and hospital management team was highly valuable in promoting open discussions of challenges faced and enabled rapid implementation of strategies that contributed to the smooth piloting of the service. Digitalizing a segment of the service relieves time and resource constraints in clinical settings, making it plausible to be embedded into the existing infrastructure and systems. This was particularly important during the COVID‐19 pandemic during which manpower shortages were overcome with technology. High digital acceptance and adoption rates propelled by COVID‐19 pandemic positioned mobile technology–enabled services as a favourable option. However, it is prudent to first assess patient's access to technology and digital literacy before offering telehealth resources to improve health outcomes.

## Author Contributions

S.M.A., J.C. and S.L.L. contributed to the conception and design of the study. S.M.A. and J.C. conducted the research, analyzed the data and drafted the first version of the manuscript. S.L.L., Y.Y.D., Y.H.C. and Q.V.Y. contributed to writing and editing the manuscript. All authors have critically reviewed the manuscript and approved the final version submitted for publication.

## Conflicts of Interest

The authors declare no conflicts of interest.

## Supporting information


Appendix S1.

## Data Availability

The data that support the findings of this study are available on request from the corresponding author. The data are not publicly available due to privacy or ethical restrictions.
