# Supplementary material for: Clinical Service Incorporating Mobile Technology on Weight Loss in Patients With Metabolic Dysfunction–Associated Steatotic Liver Disease: A Translation From Research Trial
Source: Endocrinol Diabetes Metab. 2024 Apr 29;7(3):e00485. doi: 10.1002/edm2.485 (PMC11058332; doi:10.1002/edm2.485)

**Appendices**

Table S1: Proportion of patients with ≥5% weight loss and mean decrease in AST and ALT at 6 months according to app coaching period

| **App-coaching period** | **N** | **n (%)** | **AST^1^ reduction (IU/L)**  **(mean ±SD)** | **ALT^2^ reduction (IU/L)**  **(mean ±SD)** |
| --- | --- | --- | --- | --- |
| 1 month | 85 | 14 (16.5) | 13.1 ± 24.5 | 21.6 ± 42.3 |
| 2 months | 5 | 2 (40.0) | 8.7 ± 5.7 | 18.1 ± 6.8 |
| 3 months | 7 | 3 (42.9) | 5.1 ± 18.4 | 7.9 ± 28.7 |
| 4 months | 1 | 1 (100) | 2.0 ± 0 | 7.0 ± 0 |
| 5 months | 0 | NA^†^ | NA^†^ | NA^†^ |
| 6 months or more | 11 | 6 (54.5) | 26.0 ± 28.0 | 49.7 ± 58.6 |

^†^NA: Not applicable

^1^AST: Aspartate aminotransferase

^2^ALT: Alanine aminotransferase

Table S2: Logistic regression on percentage weight loss at the end of app-coaching period

| Variable | N | <5 weight loss  n(%) | ≥5 weight loss  n(%) | Unadjusted | | Adjusted | |
| --- | --- | --- | --- | --- | --- | --- | --- |
|  |  |  |  | OR (95% CI) | p-value | OR (95% CI) | p-value |
| App-coaching period  ≥6 months  <6 months | 109 | 5(45.5)  89(90.8) | 6(54.5)  9(9.2) | 12.2 (3.0- 49.2)  1.0 | <0.001 | 11.0 (2.5- 48.3)  1.0 | 0.001 |

^a^adjusted for age, gender, ethnicity, fibrosis E score and alcohol consumption

Figure S1: Screenshots of nBuddy smartphone application (app) homepage, meal logging page and interactive chat support from left to right, respectively.


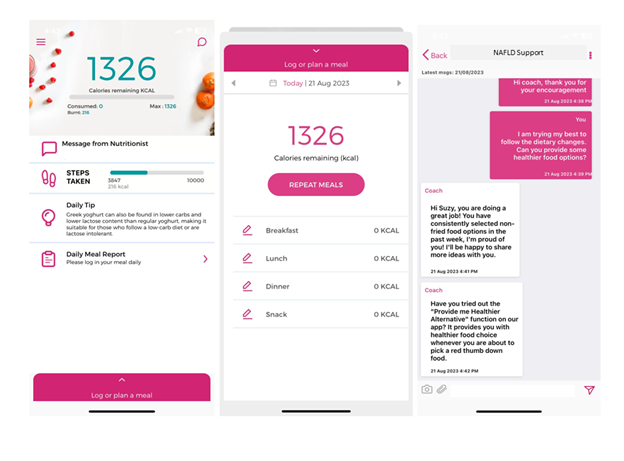

Supplement: Supplementary file 1 — Appendix S1. [file EDM2-7-e00485-s001.docx]
